# Novel Coronavirus-Induced NLRP3 Inflammasome Activation: A Potential Drug Target in the Treatment of COVID-19

**DOI:** 10.3389/fimmu.2020.01021

**Published:** 2020-05-19

**Authors:** Adnan Shah

**Affiliations:** University of Ulm, Ulm, Germany

**Keywords:** NLRP3 inflammasome, viroporins, cytokines, SARS-CoV, COVID-19

Novel coronaviruses (nCoVs) encode ion-channel proteins called viroporins such as protein E, open reading frame 3a (ORF3a) and ORF8a. These viroporins, via mechanisms such as lysosomal disruption and ion-redistribution in the intracellular environment, activate the innate immune signaling receptor NLRP3 (NOD-, LRR-, and pyrin domain-containing 3) inflammasome. This leads to the production of inflammatory cytokines such as interleukin 1β (IL-1β), IL-6 and tumor necrosis factor (TNF), causing tissue inflammation during respiratory illness caused by CoV infection. Due to this crucial role in triggering inflammatory response to infection, the NLRP3 inflammasome appears to be a potential drug target in the treatment of coronavirus disease 2019 (COVID-19), caused by SARS-CoV-2. This manuscript highlights the importance of NLRP3 inflammasome in the pathogenesis of nCoVs, discusses its known inhibitors and draws attention toward evaluation of these and similar known or novel agents for potential beneficial effects in the treatment of SARS-CoV-2 (COVID-19).

The twenty-first century has witnessed the emergence of three novel coronaviruses (nCoVs): The first outbreak was caused by severe and acute respiratory syndrome coronavirus (SARS-CoV) that emerged in Southeast China in 2002, followed by the Middle East respiratory syndrome-related coronavirus (MERS-CoV) in 2012 (1). The recent pandemic which is caused by SARS-CoV-2 originated at Wuhan city in China in late 2019, is causing a respiratory illness named as coronavirus disease 2019 (COVID-19) which is causing morbidity and mortality worldwide (2).

CoVs carry a positive-sense, single-stranded RNA genome of about 30 kb and the virion nucleocapsid is surrounded by an envelop which is studded with spike (S), membrane (M), and envelop (E) proteins (3, 4). The spike (S) glycoprotein recognizes and interacts with its target called angiotensin converting enzyme 2 (ACE2) receptor on the host cell surface, mediating viral entry during the infection cycle (5). Identifying and exploiting promising therapeutic targets has always been an area of intensive research in the treatment of viral diseases. In this respect, the spike protein of SARS-CoV is also viewed as a drug target due to its role in a crucial checkpoint of viral infection, i.e., viral attachment and entry in to the host cell. Nevertheless, there are also other virus-host interaction mechanisms (see below) that offer attractive targets for potential therapies in the context of infections caused by SARS-CoVs.

## Role of NLRP3 Inflammasome In Novel Coronavirus Pathogenesis

**Figure 1:**
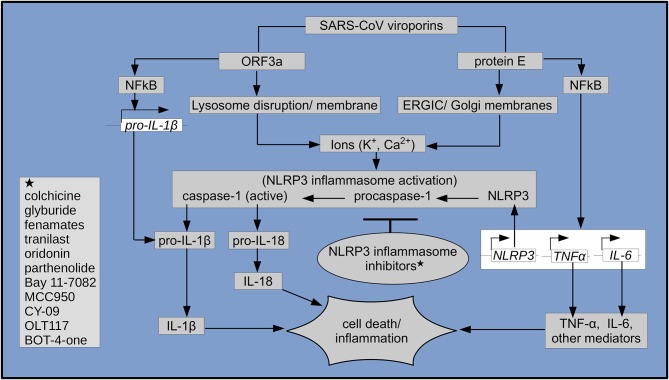
Schematic representation of SARS-CoV viroporin-mediated NLRP3 inflammasome activation, its inhibitors (shown with asterisks) and downstream inflammatory cascades leading to inflammation and cell death. Genes (italicized) in empty boxes, respective proteins in gray boxes.

Like other animal viruses, SARS-CoV also encode three ion-channel (IC) proteins called viroporins, namely the protein E, open reading frame 3a (ORF3a) and ORF8a. It has been observed that during the course of viral infections, these viroporins oligomerize and form pores, that disrupt normal physiological homeostasis in the host cell and thus contribute to the viral pathogenicity (4, 6, 7). In SARS-CoV, two of the viroporins, i.e., the more dominant protein E and also ORF3a, each carrying a PDZ-binding motif (PBM, which interacts with cellular proteins) and also having IC activity, were reported to be required for optimal viral replication. Of these, the protein E was shown to be necessary for viral virulence (8). Moreover, E protein was shown to be essential, as its absence led to the attenuation of SARS-CoV. In fact, E protein is involved in several signaling mechanisms that ultimately results in inflammation during infection. In addition to its role in activation of the inflammatory NF-kB pathway and interaction of its PBM with syntenin proteins which trigger activation of the p38 MAPK (9, 10) it also forms a calcium ion (Ca^2+^) channel in the Endoplasmic Reticulum Golgi Apparatus Intermediate Compartment (ERGIC)/Golgi membranes. As a result of this, changes in calcium homeostasis in the intracellular environment leads to activation of the cytosolic innate immune signaling receptor NLRP3 (NOD-, LRR-, and pyrin domain-containing 3) inflammasome (10), shown in Figure 1. The NLRP3 is composed of adapter component apoptosis-associated speck-like protein carrying a caspase activation and recruitment domain (ASC) and the catalytically inactive procaspase-1 (11, 12). It has been shown that several external and internal stimuli including viral RNA, activate the NLRP3 inflammasome via mechanisms such as formation of pores with ion-redistribution and lysosomal disruption, resulting in inflammation and associated cell death called pyroptosis (13). Upon activation of the NLRP3, its procaspase-1 is converted into the active effector protease caspase-1, which then causes cleavage and maturation of pro-inflammatory cytokines such as pro-interleukin 1β (pro-IL-1β) into its active form IL-1β as well as that of IL-18. These trigger a cascade of other downstream mediators of inflammation such as interleukin 6 (IL-6), tumor necrosis factor (TNF), prostaglandins and leukotrienes (13, 14). Accordingly, it was also observed that IL-1β, among other pro-inflammatory mediators, was produced in SARS-CoV infected ACE2- (viral receptor) expressing epithelial cells, pneumocytes and macrophages of bronchial and pulmonary tissues (15). In agreement with the role of E protein in triggering pro-inflammatory cytokines, it was also shown that E protein ion channel activity promote lung inflammation, fluid accumulation and bronchoalveolar epithelial damage. Further confirming this role, studies with a mutant E protein lacking IC activity showed better outcome particularly in terms of reduced edema in tissues (10, 16). Moreover, consistent with these findings, it was observed that the HIV-1 virus Vpu channel inhibitor Hexamethylene amiloride (HMA) also hindered coronavirus replication in cultured cells and inhibited the conductance of E protein ion channels in human coronavirus 229E (HCoV-229E) and mouse hepatitis virus (MHV) (17). Likewise, the ORF3a protein, a potassium (K^+^) ion channel viroporin, was shown to render host cell lysosome dysfunctional and cause caspase-1 activation either directly or via increased potassium (K^+^) efflux, leading to the NLRP3 inflammasome activation. Furthermore, it caused NFkB-mediated up-regulation of transcription of the pro-IL-1β cytokine gene and pyroptotic cell death (7, 14, 18) (see Figure 1).

Therefore, it is evident that SARS-CoV encoded viroporins, i.e., E protein and ORF3a activate the NLRP3 inflammasome and assembly. This leads to activation of inflammatory cascade involving cytokines such as IL-1β, IL-6, TNF, and other mediators as part of the host inflammatory responses to SARS-CoV infection and contribute to tissue damage.

## NLRP3 Inflammasome: A Potential Drug Target In COVID-19

Although, innate immune mechanisms such as optimal activation of the NLRP3 inflammasome plays an important role in antiviral host defenses, its aberrant activation and downstream mediators often lead to pathological tissue injury during infection (19). Also, infection with SARS-CoV is known to induce a storm of pro-inflammatory cytokines, especially IL-1β, IL-6, and TNF. These play an important role in the progression of tissue inflammation causing acute respiratory distress syndrome ARDS (10), which is a form of acute lung injury (ALI) and often leads to death. It is noteworthy that ARDS has been the leading cause of death in patients infected with SARS-CoV and MERS-CoV (1). Several studies have reported the important role of NLRP3 inflammasome activation in relation to the pathogenesis of ARDS and ALI (20–22). The pathogenesis of ARDS is driven by these pro-inflammatory cytokines, i.e., IL-1β, IL-6, and TNF and other mediators of inflammation. This is manifested by pathological events such as recruitment of inflammatory and phagocytic cells, complement activation, opsonization, increased permeability of endothelial and epithelial cells causing disruption of the air-blood barrier and accumulation of protein-rich fluid in alveoli of lungs, as well as other systemic and hemodynamic effects (23–25). Consistent with this cytokine-mediated immunopathology, elevated levels of IL-1β, IL-6, and TNF have also been observed in the broncho-alveolar lavage and plasma of ARDS patients (26). Moreover, it has been observed that there is a positive correlation between serum level of these cytokines and mortality rate in ARDS patients (27).

Based on this strong inflammatory potential of the NLRP3 inflammasome in the context of infections caused by SARS-CoVs, it appears to be an important druggable target, and its inhibition can potentially reduce tissue inflammation, also in the context of COVID-19. Based on the observed divergence of some SARS-CoV-2 encoded activators of inflammasome (viroporins) from that of SARS-CoV, comparative mechanistic studies of these viral proteins particularly in relation to NLRP3 inflammasome activation are yet to be performed. Nevertheless, cytokine storm is the main cause of inflammation in COVID-19 highlighting an important role of NLRP3 inflammasome. Accordingly, high levels of IL-1β and other cytokines have been found in COVID-19 patients (28). Whereas, a variety of drugs such as remdesivir (29), favipiravir (30), glucocorticoids (31), chloroquine (32), hydroxychloroquine plus azithromycin (2) have recently been tested for their potential beneficial effect, however, airway management and ventilatory support (33) remain the mainstay of treatment in critically ill COVID-19 patients. Given the key role of cytokines in causing inflammation, blocking their effects using biologic agents has revolutionized the treatment of rheumatoid arthritis, psoriasis, inflammatory bowel disease, and other auto-inflammatory diseases (34). Likewise, based on the SARS-CoV-2-induced cytokine-mediated inflammatory response, biologic agents that target cytokines such as the IL-1 receptor antagonist Anakinra, antibody against IL-6 receptor, i.e., Tocilizumab and anti-interferon gamma (IFN-γ) antibody Emapalumab have also been considered in clinical studies. Nevertheless, there is a dire need of effective therapy, novel agents or repurposed drugs, against the novel SARS-CoV-2 (COVID-19) so that to reduce mortality of this disease.

Efforts have been made to find potential inhibitors of the NLRP3 inflammasome, especially in the context of its role in various inflammatory diseases. Luckily, several inhibitors of the NLRP3 inflammasome including natural products as well as approved drugs, have been identified (see Figure 1). Known for their anti-inflammatory properties, natural products such as Oridonin (found in *Rabdosia rubescens* plant) and Parthenolide (sesquiterpene lactone found in *feverfew* plant) as well as synthetic compound Bay 11-7082 and related vinyl sulfone compounds have been shown to exert their effects via inhibition of the NLRP3 inflammasome. Interestingly, parthenolide and Bay 11-7082, inhibiting the NLRP3 inflammasome and inflammatory NFkB pathways, were shown to reduce lung inflammation and improve survival in SARS-CoV-infected animals (9, 35, 36).

Likewise, a sulfonylurea drug Glyburide which is widely used for the treatment of Diabetes type 2, was also shown to inhibit the NLRP3 inflammasome. Primarily acting by blocking the ATP-sensitive K^+^ channels (K_ATP_) in β-cells of the pancreas, Glyburide was shown to act upstream and prevent NLRP3 inflammasome activation. Interestingly, Glyburide-mediated inhibition of K^+^ efflux was shown to inhibit NLRP3 and secretion of IL-1β in cells infected with RNA viruses, i.e., vesicular stomatitis virus (VSV) and encephalomyocarditis virus (EMCV) (19, 37). Similarly, Tranilast, a drug used for the treatment of allergic conditions such as bronchial asthma, was shown to inhibit the NFkB pathway, several cytokines as well as the NLRP3 oligomerization, thereby preventing the inflammasome assembly. Based on these effects, Tranilast showed significant beneficial effects in animals models of NLRP3 inflammasome-related diseases of humans (38).

More importantly, an alkaloid drug Colchicine which is known for its effects such as tubulin disruption, alteration of E-selectin distribution on endothelial surfaces, inducing loss of adhesion molecule L-selectins and preventing adhesion and recruitment of neutrophil, has also been shown to inhibit activation of the NLRP3 inflammasome. Subsequently, this led to blocking of the pro-inflammatory IL-1β and IL-18 cytokine production (39, 40). Colchicin is frequently used for auto-inflammatory conditions such as gouty arthritis (41) and familial mediterranean fever (FMF) (42, 43). However, its anti-inflammatory role due to inhibition of the NLRP3 inflammasome activation, has also been shown in other conditions such as acute coronary syndrome (ACS) (44), oxidized low-density lipoprotein (oxLDL) and cholesterol crystal-induced macrophage activation (45) and non-steroidal anti-inflammatory drugs- (NSAIDs) induced small intestinal injury (46).

NSAIDs is a group of anti-inflammatory drugs, inhibiting cyclooxygenase (COX) enzymes in the synthesis of prostaglandins and other mediators, and widely used for the treatment of pain and inflammation. Studies have shown that, unlike other NSAIDs, fenamates (mefenamic acid, flufenamic acid) selectively inhibit the NLRP3 inflammasome and IL-1β release via inhibiting the membrane volume-regulated anion (Cl^−^) channel (VRAC), independent of its cyclooxygenase-1 (COX-1) mediated anti-inflammatory activity (47). In agreement with these findings, fenamates (mefenamic acid and meclofenamic acid) were observed to have considerable activity against viral replication, and a combination of ribavirin together with mefenamic acid was shown to be effective in reducing viral yield in cells infected with a positive-sense RNA genome chikungunya virus (48). Several other compounds such as MCC950 (49), CY-09 (50), OLT117 (51), and a benzoxathiole derivative BOT-4-one (52) have been shown to inhibit the NLRP3 inflammasome and have been discussed in relation to NLRP3-associated inflammatory diseases.

To summarize, this manuscript underlines the crucial role of NLRP3 inflammasome activation in the pathogenesis of diseases caused by SARS-CoVs, discusses reported inhibitors of the NLRP3 inflammasome in the context of inflammatory diseases and draws attention toward potential role of these (and similar agents) inhibitors in the treatment of SARS-CoV-2 (COVID-19). To this end, the evaluation of these reported (and other similar known or novel agents) inhibitors of the NLRP3 inflammasome in pre-clinical and/or clinical studies might offer new alternatives, especially in the form of potential repurposing of approved drugs for the treatment of COVID-19. Furthermore, considering the clinical use of several NLRP3 inhibitor drugs for the treatment of other inflammatory diseases, controlled studies of these co-morbid patients might also determine potential usefulness of these agents in the treatment of COVID-19.

## Author Contributions

AS conceived the idea, surveyed literature, and wrote the manuscript.

## Conflict of Interest

The author declares that the research was conducted in the absence of any commercial or financial relationships that could be construed as a potential conflict of interest.
